# Socioeconomic disparities in cognitive impairment, quality of life, and mortality among older adults in Germany

**DOI:** 10.1371/journal.pone.0328988

**Published:** 2025-07-28

**Authors:** Omar Hahad, Jasmin Ghaemi Kerahrodi, Isabel Heinrich, Katharina Geschke, Katja Petrowski, Elmar Brähler, Julia Petersen, Anna C. Reinwarth, Julian Chalabi, Alexander K. Schuster, Emilio Gianicolo, Karl Lackner, Peter R. Galle, Stavros Konstantinides, Sadeer Al-Kindi, Philipp Wild, Oliver Tüscher, Matthias Michal, Manfred E. Beutel

**Affiliations:** 1 Department of Cardiology – Cardiology I, University Medical Center of the Johannes Gutenberg-University Mainz, Mainz, Germany; 2 German Center for Cardiovascular Research (DZHK), Partner Site Rhine-Main, Mainz, Germany; 3 Department of Psychosomatic Medicine and Psychotherapy, University Medical Center of the Johannes Gutenberg-University Mainz, Mainz, Germany; 4 Department of Psychiatry and Psychotherapy, University Medical Center of the Johannes Gutenberg-University Mainz, Mainz, Germany; 5 Medical Psychology and Medical Sociology, University Medical Center of the Johannes Gutenberg-University Mainz, Mainz, Germany; 6 Preventive Cardiology and Preventive Medicine, Department of Cardiology, University Medical Center of the Johannes Gutenberg-University Mainz, Mainz, Germany; 7 Department of Ophthalmology, University Medical Center of the Johannes Gutenberg-University Mainz, Mainz, Germany; 8 Institute of Medical Biostatistics, Epidemiology & Informatics, University Medical Center of the Johannes Gutenberg-University Mainz, Mainz, Germany; 9 Institute of Clinical Physiology, National Research Council, Lecce, Italy; 10 Institute of Clinical Chemistry and Laboratory Medicine, University Medical Center of the Johannes Gutenberg-University Mainz, Mainz, Germany; 11 Department of Internal Medicine I, University Medical Center of the Johannes Gutenberg-University, Mainz, Germany; 12 Center for Thrombosis and Hemostasis (CTH), University Medical Center of the Johannes Gutenberg-University Mainz, Mainz, Germany; 13 Division of Cardiovascular Prevention and Wellness, Department of Cardiology, DeBakey Heart & Vascular Center, Houston Methodist, Houston, Texas, United States of America; 14 Institute for Molecular Biology, Mainz, Germany; 15 Department of Psychiatry, Psychotherapy and Psychosomatic Medicine, University Medicine Halle (Saale) of the Martin Luther University Halle-Wittenberg, Halle (Saale), Germany; 16 Leibniz Institute for Resilience Research (LIR), Mainz, Germany; Queen's University Belfast, UNITED KINGDOM OF GREAT BRITAIN AND NORTHERN IRELAND

## Abstract

**Background:**

The global older population is increasing, leading to a rise in non-communicable diseases and disabilities, particularly in Western countries. With the aging population expanding and the number of older adults with cognitive impairments expected to rise, there is increasing interest in understanding the socioeconomic disparities associated with cognitive impairment. This study investigates the relationships between socioeconomic status (SES), cognitive impairment, quality of life, and mortality among older adults in Germany.

**Methods:**

Data from the senior cohort (*N* = 1,069) of the German Gutenberg Health Study (2017–2024) were analyzed, focusing on older adults aged 75–85 years. Regression modeling with sequential adjustment for covariates was employed to determine the association between various domains of SES (SES index comprising educational background, occupational status, and household net-income) and cognitive impairment (Montreal Cognitive Assessment), quality of life (EUROHIS-QOL), and all-cause mortality.

**Results:**

Cognitive impairment scores varied significantly by SES with higher SES being associated with better cognitive performance. Among the SES domains, the household net-income score was the strongest predictor of cognitive impairment. Likewise, higher SES was significantly associated with higher quality of life, whereas no association between cognitive impairment and quality of life was found. Additionally, cognitive impairment was significantly associated with higher all-cause mortality, whereas SES did not show a significant association with mortality. No significant interactions between SES and cognitive impairment were observed in relation to quality of life or all-cause mortality.

**Conclusion:**

Among older adults, SES is strongly associated with cognitive impairment. However, cognitive impairment emerges as a more significant risk factor for all-cause mortality than SES. These findings suggest the need for public health strategies to prioritize cognitive health monitoring and targeted interventions, while simultaneously addressing social inequalities, to reduce the burden of these adverse outcomes.

## Introduction

The global older population is increasing, leading to a rise in the prevalence of non-communicable diseases and disabilities [1]. Population projections for Germany indicate a continued increase in the number and proportion of people aged 65 and above. By 2040, the share of people aged 65 or older is expected to reach almost 28% of the general population [2]. This trend is projected to continue, with the proportion of people aged 60 and over estimated to reach 35% by 2030 and 38% by 2050 [3]. Cognitive impairment is a significant risk factor for poor health and increased morbidity among older adults, and it contributes to a substantial burden on public health systems [4]. Better cognitive performance is associated with improved mental [5] and physical health [6], quality of life [7], and reduced mortality [8]. Currently, it is estimated that approximately 55 million individuals worldwide are living with dementia, defined as a severe decline in cognitive performance impairing daily life and independent functioning, with over 60% residing in low- and middle-income countries [9]. By the end of 2021, about 1.8 million people in Germany were living with dementia, with 360,000–440,000 new cases that year. Projections for 2033 estimate that 1.65 to 2 million individuals aged 65 and above could be affected in Germany [10]. Socioeconomic disparities play a crucial role in cognitive decline with its impact being evident in various aspects, including clinical severity, neurodegeneration, altered brain dynamics, heightened proteinopathy, and allostatic overload [11]. A fundamental approach to examining socioeconomic disparities at the individual level is the socioeconomic status (SES). The SES refers to an individual or group’s standing within the socioeconomic spectrum, determined by factors like income, education, and occupation [12]. Among the various socioeconomic factors, education is the most extensively studied, showing protective effects on cognitive performance across different cultural and geographic contexts [13]. However, fewer studies have examined the simultaneous influence of other socioeconomic factors such as income, wealth, and occupation on cognitive performance in older adults, with inconsistent findings further complicating our understanding of their impact. Interestingly, a recent meta-analysis on education and age-related decline in cognitive performance found no evidence for a consistent and substantial association between educational attainment and changes in cognitive performance in the general population [14].

With the aging population and the expected increase in older adults with cognitive impairment, there is a growing interest in exploring the relationship between SES and cognitive impairment. Despite the well-established link between SES and cognitive impairment, evidence from high-income countries with universal healthcare systems remains limited, particularly when using multidimensional assessments of SES that simultaneously consider education, occupation, and household net-income. Furthermore, few studies have jointly investigated the impact of SES on cognitive impairment, quality of life, and mortality within the same aging population. Thus, this study aims to provide added value by offering a comprehensive analysis of these associations in a large, well-characterized sample of older adults in Germany.

## Methods

### The Gutenberg Health Study

The Gutenberg Health Study (GHS) is a comprehensive, prospective, observational cohort study based at the University Medical Center in Mainz, Germany, which has been described recently in detail [15]. The current analysis utilized data from the GHS, which involved a randomly selected sample from the local registries of Mainz city and the Mainz-Bingen district. The study included 15,010 participants at baseline, aged 35–74 years. Baseline data collection occurred from April 2007 to April 2012, with follow-up assessments conducted at 5- and 10-years post-enrollment (2012–2017 and 2017–2022, respectively), using similar procedures as the baseline. In addition to the core cohort, new participants aged 25–44 years (young cohort) and 75–85 years (senior cohort) were recruited at the 10-year follow-up, including the implementation of cognitive impairment assessments [16]. Thus, all participants included in this analysis were recruited between 2017 and 2022 as part of the senior cohort study.

The study’s protocol and documentation were approved by the ethics committee of the Medical Chamber of Rhineland-Palatinate (reference number 837.020.07(5555)), and by both local and federal data protection authorities. Exclusion criteria included insufficient German language skills and psychological or physical conditions that would interfere with participation. The study was conducted in accordance with the revised Helsinki protocol and the guidelines for Good Clinical and Epidemiological Practice. All participants provided written informed consent before taking part in the study.

### Socioeconomic status

The assessment of SES has recently been described in detail [12]. Briefly, the SES was evaluated using a validated questionnaire (SES index) administered through a computer-assisted interview [17,18]. The SES index incorporates scores from three domains: educational background, occupational status, and household net-income. Occupational status referred to the occupation held during the working life. Household net-income includes pensions, part-time jobs, rental income, capital gains, social assistance, and any other regular monthly earnings. Each domain was rated on a scale from 1 to 7. Consequently, the SES index total score could range from 3 to 21, with 21 representing the highest SES and 3 the lowest. For clarity and comparability, the SES index total score was categorized into three groups: 3 to 7.7, 7.8 to 14.1, and 14.2 to 21, which correspond to low, medium, and high SES, respectively, following published guidelines [17,18].

### Cognitive impairment

Cognitive impairment was assessed using the paper-and pencil version of the Montreal Cognitive Assessment (MoCA, version 8.1 in German), a widely used brief cognitive screening tool. Due to its high sensitivity and specificity the MoCA is frequently employed to detect cognitive impairment [19,20]. The evaluation generates a total score and assesses the following cognitive domains: memory, executive functions, attention, language, visuospatial abilities, and orientation. The original normative data were published in 2005 and were developed on a modest sample of 90 healthy controls, 94 individuals with mild cognitive impairment, and 93 individuals with Alzheimer’s disease [19]. A total score of 26 out of 30 points or above is indicative of normal cognitive functioning (with a specificity of 87%), whereas a score below this threshold is suggestive of mild cognitive impairment. It is validated for individuals aged 55–85 [19,20].

### Quality of life

The EUROHIS-QOL is an 8-item self-report tool developed from the WHOQOL-100 and WHOQOL-BREF. It assesses four domains of quality of life: physical, psychological, social, and environmental, with items addressing overall quality of life, general health, energy levels, daily activities, self-esteem, social relationships, financial status, and home environment. The questionnaire focuses on evaluating general quality of life. Responses are given on a 5-point Likert scale, with higher total scores reflecting better quality of life. Psychometric evaluations from the initial pilot study and the later field study showed strong reliability [21]. A cross-cultural study found a satisfactory internal consistency with a Cronbach’s alpha of 0.83 [22], and later studies using the German version of the questionnaire reported appropriate psychometric properties [23,24].

### Mortality

Mortality updates were conducted every three months through systematic inquiries to the registry offices and the Rhineland-Palatinate mortality registry. To facilitate thorough death reviews, official death certificates were obtained for each case. This process ensured that all relevant mortality data was accurately recorded and analyzed.

### Covariates

Somatic diseases and comorbidities were identified through the analysis of medical records, which included diagnoses from physicians or those made during study visits. This review covered a range of conditions, such as coronary artery disease, myocardial infarction, stroke, chronic heart failure, diabetes mellitus, and chronic obstructive pulmonary disease. Participants were classified based on their smoking status as either current smokers or non-smokers, with non-smokers encompassing both individuals who had never smoked and those who had quit [25]. Arterial hypertension was identified if participants were on antihypertensive medication or exhibited a resting systolic blood pressure of 140 mmHg or higher, or a diastolic blood pressure of 90 mmHg or higher, based on average measurements from the second and third readings after 8 and 11 minutes of rest. Obesity was defined as having a body mass index of 30 kg/m^2^ or higher [26]. Dyslipidemia was diagnosed based on a physician’s diagnosis, a LDL to HDL cholesterol ratio exceeding 3.5, or triglyceride levels of 150 mg/dL or higher. A family history of myocardial infarction or stroke was recorded if a first-degree relative (female ≤ 65 years or male ≤ 60 years) had experienced such events. For alcohol consumption exceeding recommended limits, participants were categorized into “below” or “above recommended limit” based on self-reported intake, with cut-off values set at > 24 g per day for men and > 12 g per day for women. Hearing impairment was defined as a hearing threshold of 20 dB or more [27]. Depression was identified using the Patient Health Questionnaire-9 (PHQ-9) total score of 10 or higher [25].

### Statistical analysis

Participants with missing SES index information (*n* = 459) were excluded from the analysis. Therefore, the study sample includes only participants from the senior cohort who completed the MoCA and had complete SES index data. The study sample’s characteristics were stratified by SES groups (low, medium, and high), with categorical variables presented as absolute and relative frequencies, and continuous variables shown as mean values with standard deviations. For variables with skewness greater than 1, the median (Q1, Q3) was reported. Statistical comparisons between SES groups were conducted using the *t* trend test. The associations between SES index total score, its domain scores (education, occupation, and household net-income adjusted for each other, per point increase), and SES groups (low as the reference category, medium, and high) with MoCA total scores were analyzed using linear regression models. Beta estimates and 95% confidence intervals (CI) were reported. Furthermore, we explored the relationship between the SES index total score and MoCA total score with quality of life (EUROHIS-QOL total score), testing for interaction effects via an interaction term using linear regression modeling. To investigate the association between SES index total score and MoCA total score on all-cause mortality risk, we employed Cox proportional hazards survival regression models with hazard ratios and 95% CI, as well as Kaplan-Meier analysis with log rank testing. The regression models were adjusted sequentially. Model 1 included adjustments for sex (binary) and age (continuous). Model 2 further adjusted for alcohol consumption (binary), depression (binary), arterial hypertension (binary), current smoking (binary), obesity (binary), dyslipidemia (binary), and family history of myocardial infarction or stroke (binary). Model 3 additionally accounted for prevalent comorbidities (a composite of coronary artery disease, myocardial infarction, stroke, chronic heart failure, diabetes mellitus, or chronic obstructive pulmonary disease). To avoid a substantial reduction in sample size, we decided not to include hearing impairment as a covariate. This decision was based on the absence of significant differences in the prevalence of hearing impairment between SES groups and the results of a sensitivity analysis, which showed no substantial differences when adjusting for hearing impairment. In this study, *p* values are reported precisely and should be interpreted as a continuous measure of statistical strength of an association, with *p* values <0.05 considered significant. All statistical analyses were performed using R software version 4.2.1 (http://www.r-project.org/).

## Results

### Sample characteristics

**Table 1** presents the characteristics of the senior cohort from the GHS stratified by SES groups (*N* = 1,069). Women were more prevalent in the low and medium SES groups (55.2% and 52.5%, respectively) compared to the high SES group (21.8%, *p* for trend <0.0001). Cognitive impairment scores varied significantly by SES. The mean MoCA total score was lowest in the low SES group (17.5 ± 7.5) and highest in the high SES group (22.8 ± 5.8) (*p* for trend <0.0001). Quality of life, measured by the EUROHIS-QOL total score, was significantly higher in participants with higher SES (33.5 ± 3.5 in high SES vs. 31.9 ± 3.3 in low SES, *p* for trend <0.0001). The prevalence of diabetes mellitus and obesity decreased significantly with increasing SES, whereas alcohol consumption above the recommended limit was more common in the high SES group.

**Table 1:** Characteristics of the senior cohort from the Gutenberg Health Study (2017-2022) stratified by socioeconomic status (SES) groups (*N* = 1,069).

	Low SES (*n *= 116)	Medium SES (*n *= 613)	High SES (*n *= 340)	*P* for trend
Sex (women) – % (no.)	55.2 (64)	52.5 (322)	21.8 (74)	**<0.0001**
Age (years) – mean ± SD	79.1 ± 2.8	78.9 ± 2.7	79.1 ± 2.6	0.40
*Socioeconomic status (SES)*				
SES index total score (3–21) – mean ± SD	6.13 ± 0.82	11.07 ± 1.95	17.82 ± 2.04	**<0.0001**
Education score (1-7) – mean ± SD	1.74 ± 0.48	2.45 ± 0.93	5.65 ± 1.48	**<0.0001**
Occupation score (1-7) – mean ± SD	2.01 ± 0.86	3.95 ± 1.29	5.78 ± 1.13	**<0.0001**
Household net-income score (1-7) – mean ± SD	2.40 ± 0.81	4.71 ± 1.27	6.31 ± 0.81	**<0.0001**
*Cognitive impairment (MoCA)*				
Cognitive impairment (MoCA) total score – mean ± SD	17.48 ± 7.54	20.56 ± 6.65	22.76 ± 5.77	**<0.0001**
*Hearing impairment*				
< 20 dB vs. ≥ 20 dB – % (no.)	88.1 (52)	87.8 (351)	85.7 (192)	0.47
*Quality of life*				
Quality of life (EUROHIS-QOL) total score – mean ± SD	31.9 ± 3.3	32.4 ± 3.3	33.5 ± 3.5	**<0.0001**
*Risk factors and comorbidities*				
Current smoking – % (no.)	2.8 (3)	3.2 (19)	3.4 (11)	0.75
Depression (PHQ-9 ≥ 10) – % (no.)	4.3 (5)	2.8 (17)	1.8 (6)	0.13
Alcohol consumption above recommended limit – % (no.)	16.4 (19)	18.4 (113)	25.0 (85)	**0.012**
Diabetes mellitus – % (no.)	27.8 (32)	16.6 (102)	13.5 (46)	**0.0015**
Arterial hypertension – % (no.)	81.9 (95)	79.0 (484)	76.2 (259)	0.16
Obesity – % (no.)	30.2 (35)	26.1 (160)	15.0 (51)	**<0.0001**
Dyslipidemia – % (no.)	57.4 (66)	44.9 (275)	45.4 (154)	0.11
Family history of myocardial infarction or stroke – % (no.)	6.0 (7)	4.7 (29)	5.3 (0.94)	0.94
Any cardiovascular disease – % (no.)	24.3 (28)	19.7 (119)	22.9 (76)	0.81
Coronary artery disease – % (no.)	3.5 (4)	4.0 (24)	6.2 (20)	0.14
Myocardial infarction – % (no.)	4.3 (5)	3.0 (18)	2.7 (9)	0.45
Stroke – % (no.)	7.8 (9)	5.3 (32)	4.5 (15)	0.21
Chronic heart failure – % (no.)	2.6 (3)	4.3 (26)	0.9 (3)	0.056
Chronic obstructive pulmonary disease – % (no.)	7.8 (9)	6.7 (41)	5.0 (17)	0.22

Continuous variables are shown as mean and standard deviation or if |skewness| > 1 by median (Q1, Q3). Binary variables are described as relative and absolute frequencies. *P* for trend is calculated by *t t*rend test.

Socioeconomic status (SES) total score ranges from 3 to 21 with 21 indicating the highest SES and 3 the lowest SES.

SES domain scores (i.e., education, occupation, and household net-income) range from 1 to 7 with 7 indicating the highest value and 1 the lowest value.

Montreal Cognitive Assessment (MoCA): a total possible score of 30 points, a score of 25 or less is indicative of cognitive impairment.

Hearing thresholds below 20 dB are classified as normal.

Patient Health Questionnaire-9 (PHQ-9) total score of 10 or higher is used to identify cases of depression. The total score in the PHQ-9 ranges from 0 to 27.

Alcohol consumption above recommended limit denotes >24 g per day for men and >12 g per day for women.

### Socioeconomic status and cognitive impairment

**Tables 2** shows the associations between SES and cognitive impairment. Higher SES was significantly positively associated with higher MoCA total scores (beta 0.346, 95% CI 0.248–0.445, *p* < 0.0001 in model 3). Among the SES domains, the household net-income score was the strongest predictor of cognitive impairment with the highest beta estimates across all models.

**Table 2:** Cross-sectional association analysis between socioeconomic status and cognitive impairment (MoCA total score) (data from the Gutenberg Health Study 2017-2022).

	*N*	Model 1		Model 2		Model 3	
Beta estimate [95% CI]	*P* value	Beta estimate [95% CI]	*P* value	Beta estimate [95% CI]	*P* value
SES index total score	1,001	0.384 [0.289; 0.479]	**<0.0001**	0.347 [0.249; 0.446]	**<0.0001**	0.346 [0.248; 0.445]	**<0.0001**
Education score	1,001	0.681 [0.472; 0.891]	**<0.0001**	0.592 [0.377; 0.806]	**<0.0001**	0.574 [0.359; 0.789]	**<0.0001**
Occupation score	842	0.697 [0.435; 0.959]	**<0.0001**	0.661 [0.395; 0.927]	**<0.0001**	0.680 [0.414; 0.946]	**<0.0001**
Household net-income score	989	0.806 [0.548; 1.064]	**<0.0001**	0.743 [0.479; 1.007]	**<0.0001**	0.756 [0.491; 1.020]	**<0.0001**
Medium SES (vs. low)	1,001	3.029 [1.747; 4.311]	**<0.0001**	2.723 [1.408; 4.037]	**<0.0001**	2.829 [1.516; 4.143]	**<0.0001**
High SES (vs. low)	1,001	5.291 [3.902; 6.679]	**<0.0001**	4.711 [3.280; 6.142]	**<0.0001**	4.783 [3.353; 6.213]	**<0.0001**

Beta estimates and 95% CI are derived from a linear regression model modeling for cognitive impairment (MoCA total score) disease with socioeconomic status being the independent variable. *N* denotes model 3.

Model 1 was adjusted for sex and age.

Model 2 was additionally adjusted for alcohol consumption, depression, arterial hypertension, current smoking, obesity, dyslipidemia, and family history of myocardial infarction or stroke.

Model 3 was additionally adjusted for prevalent comorbidities (comprising coronary artery disease, myocardial infarction, stroke, chronic heart failure, diabetes mellitus, or chronic obstructive pulmonary disease.

### Socioeconomic status, cognitive impairment, and quality of life

**Table 3** display the association between SES, cognitive impairment, and quality of life. The SES index total score was significantly associated with quality of life (*p* = 0.00018 in model 3), but cognitive impairment was not (*p* = 0.23). There was no significant interaction between SES and cognitive impairment on quality of life (*p* = 0.22).

**Table 3:** Cross-sectional association analysis between socioeconomic status (SES index total score), cognitive impairment (MoCA total score), and quality of life (EUROHIS-QOL total score) (data from the Gutenberg Health Study 2017-2022).

	*N*	Model 1		Model 2		Model 3	
Beta estimate [95% CI]	*P* value	Beta estimate [95% CI]	*P* value	Beta estimate [95% CI]	*P* value
SES (SES index total score)	922	0.133 [0.079; 0.187]	**<0.0001**	0.115 [0.059; 0.170]	**<0.0001**	0.106 [0.051; 0.161]	**0.00018**
Cognitive impairment (MoCA total score)		0.020 [−0.018; 0.059]	0.30	0.024 [−0.015; 0.063]	0.22	0.024 [−0.015; 0.062]	0.23
Interaction SES (SES index total score) and cognitive impairment (MoCA total score)		0.006 [−0.002; 0.013]	0.14	0.005 [−0.003; 0.013]	0.20	0.005 [−0.003; 0.013]	0.22

Beta estimates and 95% CI are derived from a linear regression model modeling for quality of life (EUROHIS-QOL total score) disease with socioeconomic status (total score) and cognitive impairment (MoCA total score) being the independent variables. *N* denotes model 3.

Model 1 was adjusted for sex and age.

Model 2 was additionally adjusted for alcohol consumption, depression, arterial hypertension, current smoking, obesity, dyslipidemia, and family history of myocardial infarction or stroke.

Model 3 was additionally adjusted for prevalent comorbidities (comprising coronary artery disease, myocardial infarction, stroke, chronic heart failure, diabetes mellitus, or chronic obstructive pulmonary disease.

### Socioeconomic status, cognitive impairment, and all-cause mortality

**Fig 1:**
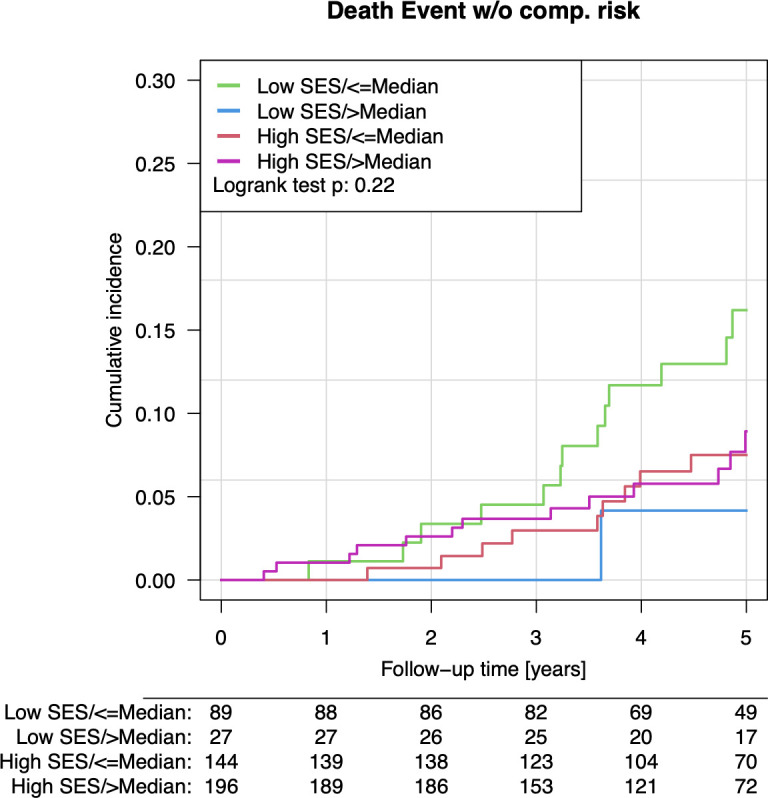
Kaplan-Meier curves illustrating the association between socioeconomic status groups (SES; low and high), cognitive impairment (MoCA total score: below or equal to vs. above the median), and the risk of all-cause death in the Gutenberg Health Study (2017-2024). The *p* value corresponds to the log rank test.

**Fig 1 and Table 4** provide the results of the association between SES, cognitive impairment, and all-cause mortality. Cognitive impairment was significantly associated with higher all-cause mortality (HR 0.953, 95% CI 0.920–0.987, *p* = 0.0067 in model 3), while SES was not significantly associated with mortality (*p* = 0.46). There was no significant interaction between SES and cognitive impairment in predicting all-cause mortality (*p* = 0.81).

**Table 4:** Longitudinal association analysis between socioeconomic status (SES index total score), cognitive impairment (MoCA total score), and all-cause mortality (data from the Gutenberg Health Study 2017-2024).

	*N*	Model 1		Model 2		Model 3	
Hazard ratio [95% CI]	*P* value	Hazard ratio [95% CI]	*P* value	Hazard ratio [95% CI]	*P* value
SES (SES index total score)	1,001	0.975 [0.924; 1.030]	0.37	0.976 [0.921; 1.035]	0.42	0.978 [0.923; 1.037]	0.46
Cognitive impairment (MoCA total score)		0.949 [0.919; 0.981]	**0.0020**	0.948 [0.916; 0.981]	**0.0025**	0.953 [0.920; 0.987]	**0.0067**
Interaction SES (SES index total score) and cognitive impairment (MoCA total score)		1.001 [0.995; 1.007]	0.71	1.001 [0.994; 1.007]	0.85	1.001 [0.994; 1.007]	0.81

Hazard ratios and 95% CI are derived from a Cox proportional hazard regression model modeling for all-cause mortality (dependent variable) with socioeconomic status (total score) and cognitive impairment (MoCA total score) being the independent variables). *N* denotes model 3 and number of deaths was 84.

Model 1 was adjusted for sex and age.

Model 2 was additionally adjusted for alcohol consumption, depression, arterial hypertension, current smoking, obesity, dyslipidemia, and family history of myocardial infarction or stroke.

Model 3 was additionally adjusted for prevalent comorbidities (comprising coronary artery disease, myocardial infarction, stroke, chronic heart failure, diabetes mellitus, or chronic obstructive pulmonary disease.

## Discussion

This study investigated the associations between SES, cognitive impairment, quality of life, and all-cause mortality among older adults in Germany, using data from the senior cohort of the GHS. The findings indicate that higher SES is significantly associated with better cognitive performance and higher quality of life. All three domains of the SES, i.e., education, occupation, and income, were found to contribute to cognitive impairment, with income exhibiting the strongest association. Cognitive impairment was linked to increased all-cause mortality, whereas SES did not show a direct association with mortality rates.

The positive association between higher SES and higher levels of cognitive performance aligns with the previous evidence, suggesting that individuals with greater socioeconomic resources may have better access to health-promoting behaviors, cognitive-stimulating activities, and healthcare services. This relationship is likely mediated through multiple pathways, including enhanced access to nutrition, education, social engagement, and healthcare, which are all crucial for maintaining cognitive performance and overall health throughout lifespan [12]. An analysis of 1,313 older adults in the US National Health and Nutrition Examination Survey (NHANES) found that higher SES and healthier lifestyles were both independently associated with better cognitive performance, while no significant interaction between SES and healthy lifestyle was observed [28]. Interestingly, a study among 773 older adults in northern Italy demonstrated that the cognitive benefits of a healthy lifestyle were most pronounced in those with low SES, highlighting the role of active lifestyles in mitigating socioeconomic health disparities in cognitive performance [29]. Meta-analytic results from 39 prospective studies involving nearly 1.5 million individuals investigating the impact of SES on cognitive impairment and dementia revealed that a low SES was associated with a 31% (RR 1.31, 95% CI 1.16–1.49) higher combined risk of cognitive impairment and dementia [30]. Stratified analyses by education, income, and occupation indicated that both low-education (RR 1.21, 95% CI 1.04–1.41) and low-income (RR 1.22, 95% CI 1.10–1.35) participants were at increased combined risk of cognitive impairment and dementia. In support of these results, the findings of a Chinese study indicated that higher SES was significantly associated with better cognitive performance in older adults, while both emotional and financial support moderated this relationship [31]. Data from 9,176 older adults in India found that education, wealth, and psychological health accounted for most of the socioeconomic inequality in cognitive impairment [32]. In contrast, a meta-analysis of 92 studies showed that the association between education and cognitive decline in older adults was generally negligible [14]. However, it should be acknowledged that there was substantial heterogeneity among the studies, which remained largely unexplained by factors such as mean age, mean educational attainment, and Gross Domestic Product per capita.

Our results align with prior research indicating that the SES is a significant predictor of cognitive performance in older adults after comprehensive adjustment for covariates. Specifically, our data show that SES as a composite factor, as well as its individual domains, i.e., education, occupation, and household net-income, affect cognitive performance. Notably, the household net-income had a stronger impact on cognitive performance compared to education and occupation (Table 2). Furthermore, our study extends these findings by demonstrating that socioeconomic disparities also influence quality of life in older adults. Additionally, cognitive impairment was found to be a more robust predictor of all-cause mortality than SES (Table 4). The age range of 75–85 years was selected as cognitive impairment becomes more pronounced with mild cognitive impairment more prevalent. While higher SES was associated with better cognitive performance, it did not predict mortality, whereas cognitive impairment was a stronger determinant, likely reflecting underlying vulnerabilities such as comorbidities. The stronger association between cognitive impairment and mortality may suggest mechanisms like impaired self-care and reduced treatment adherence. In our sample, cognitive impairment did not impact quality of life, which may be influenced more by physical and social factors than by early cognitive impairment. However, direct comparison with previous studies is challenging due to variations in definitions and measurement tools for SES, cognitive impairment, and quality of life. Differences in the study context, such as Germany’s high-income setting with universal healthcare access, as well as other methodological variations including covariate adjustment strategies, further complicate such comparisons.

The particularly strong predictive power of household net-income on cognitive performance suggests that financial stability plays a crucial role in enabling older adults to engage in activities that protect and enhance cognitive performance. Financial resources can enhance access to healthcare services, including preventive measures and early interventions for cognitive impairment, potentially improving cognitive performance. This is especially important in high-income countries like Germany, where poverty and food insecurity, especially in older adults, are becoming growing problems that negatively affect both mental and physical health [33]. For instance, a short-term supplemental income intervention for Mexican individuals aged 70 and above was shown to improve cognitive performance with the effect mediated through improved food security and healthcare access [34]. In this context, findings from Montano offer a broader life-course perspective, suggesting that the relationship between SES and health is dynamic over time, with well-being exerting a stronger influence on income than vice versa, and education being a more consistent predictor of mortality in younger and midlife adults [35]. In contrast, our findings in older adults highlight household net-income as the SES domain most strongly associated with cognitive impairment. These differences may reflect age-related shifts in the relevance of SES domains, whereby income may become more critical in late life due to its immediate impact on access to care and living conditions. Moreover, Robitaille et al. used longitudinal data from older adults to model cognitive state transitions and life expectancy [36]. They found education to be a consistent predictor of cognitive decline and mortality. These differences may reflect also variations in outcome definitions and analytical approaches. The absence of an SES-mortality association overserved in our study could reflect the moderating role of universal healthcare in Germany, which may reduce SES-related disparities in mortality. In contrast, cognitive impairment may act as a more proximal indicator of biological aging and mortality risk, capturing cumulative health burden more directly than SES.

This study leverages a large sample from the GHS, enhancing the reliability and generalizability of its findings. By using comprehensive SES domains that include individual information on educational background, occupational status, and household net-income, the study provides a nuanced understanding of the interplay SES, cognitive impairment, quality of life, and mortality including comprehensive adjustment for covariates. Despite the strengths of this study, several limitations need to be considered. The observational design limits the ability to infer causality. In particular, the association between SES, cognitive performance, and quality of life is based on cross-sectional data, which further restricts causal interpretation. Reverse causation cannot be excluded, for instance, cognitive impairment may itself influence occupational status or reduce household net-income in later life, potentially biasing the observed associations. While we adjusted for numerous potential confounders, residual confounding cannot be entirely ruled out. While validated questionnaires were used, reliability is also influenced by individuals recall accuracy and adherence. Additionally, the SES index assigns equal weight to education, occupation, and household net-income, although these domains may differ in their stability and relevance across the life course. For example, education is typically fixed in early adulthood, whereas household net-income may fluctuate considerably in older age, which may influence the interpretation of SES-related findings. Lastly, our SES measure is based in part on pre-retirement occupation, which may not fully capture late-life socioeconomic conditions. Factors such as savings, debt burden, or housing tenure, often more directly reflective of financial security in older age, were not available but could offer additional insight into socioeconomic stratification in aging populations.

The implications of these findings are substantial for public health policy and intervention strategies. Addressing socioeconomic disparities could be a critical step in mitigating cognitive impairment and improving the quality of life among older adults. Strategies to improve cognitive performance should include regular cognitive screenings and interventions tailored to at-risk populations, particularly those with lower SES. These interventions could include cognitive training programs, community engagement activities, and comprehensive healthcare services that address both physical and cognitive health needs. Future research should prioritize longitudinal studies that can more robustly examine causal relationships between SES and cognitive performance. Such studies could help identify critical periods in the life course where interventions might be most effective. Also, a better understanding of whether the effects are primarily driven by educational opportunities, access to healthcare, lifestyle factors, or other mediating variables will be crucial for designing targeted interventions. Investigating the role of chronic stress, social support, and environmental factors in this context could also yield valuable insights.

In conclusion, this study adds to the growing body of evidence highlighting the critical role of SES in cognitive performance among older adults. Higher SES is associated with better cognitive performance and quality of life, while cognitive impairment is a significant predictor of increased mortality. These findings underscore the importance of addressing socioeconomic inequalities to improve cognitive health outcomes and enhance the quality of life in the aging population.
